# Developing Tools to Assess Airborne Cyanobacterial Toxins in Southwest Florida, USA

**DOI:** 10.3390/toxins18070309

**Published:** 2026-07-16

**Authors:** James S. Metcalf, Manuel Aparicio, Sandra A. Banack, Jason Pim, John R. Cassani, Paul A. Cox

**Affiliations:** 1 Brain Chemistry Labs, P.O. Box 3464, Jackson, WY 83001, USApaul@ethnomedicine.org (P.A.C.); 2 Department of Biological Sciences, Bowling Green State University, Bowling Green, OH 43403, USA; 3 Calusa Waterkeeper, P.O. Box 1165, Fort Myers, FL 33902, USAjcass927@gmail.com (J.R.C.)

**Keywords:** airborne, toxin, HAB, cyanobacteria, risk assessment, human health

## Abstract

Cyanobacterial and harmful algal blooms are common components of the waters in and around Southwest Florida and are sufficiently frequent to be of concern with respect to adverse effects on short- and long-term human and animal health. Currently, the contribution of airborne exposure to cyanobacterial toxins is not as advanced as for the other known human exposure routes such as consumption of contaminated water or fish. An airborne monitoring device named Airborne Detection for Algae Monitoring (ADAM) was developed to collect air samples in the proximity of algal and/or cyanobacterial blooms to examine the relationship between naturally occurring toxins in the air and those in proximal Floridian waters. Twenty-one air and water samplings were performed between July and December 2021 using a filter and impinger system for airborne components, along with a water sample from the same location. Samples were assessed for microcystins, anatoxin-a, cylindrospermopsin, saxitoxin and BMAA and isomers. Furthermore, as *Karenia brevis* is common in this area, brevetoxins were also assessed in air samples. Although very few large-scale cyanobacterial bloom events were observed at sampling locations, cyanobacterial and algal toxins were present in the majority of samples at low concentration. Data obtained from ADAM indicate that people may be chronically exposed to low concentrations of cyanobacterial toxins and that further assessment is required to help protect human health.

## 1. Introduction

A plethora of toxic compounds are produced by cyanobacteria/Cyanoprokaryota [1,2,3], possessing many different modes of action such as hepatotoxicity, neurotoxicity and cytotoxicity as examples [4]. Furthermore, many groups of marine microalgae, including cyanobacteria, diatoms and dinoflagellates, can add to these cocktails [5,6,7]. Exposure to individual toxins or combinations has the potential to cause severe acute toxicity, and increasingly exposure is being linked to long-term adverse health outcomes [8,9]. To completely understand the risks to human health, an examination of the contributions from all exposure routes is necessary. Documented routes include drinking water, recreational water and consumption of contaminated food such as fish and shellfish [4,10,11,12]. Presently, the airborne exposure route is being identified as a potential significant contributor to chronic exposure, with a number of studies having already identified microcystins [13,14,15,16], anatoxin-a [17] and BMAA [18,19,20] in collected air samples, as examples. Their presence in air is likely as a result of buoyancy and wave action, resulting in bubble bursting processes and generation of lake spray aerosols (LSA) [6,21]. Consequently, a greater understanding of the type and concentration of harmful cyanobacterial and algal bloom toxins in air is necessary to better protect human and animal health [22,23,24].

Comparisons of acute toxicities through intraperitoneal and via oral administration have shown that cyanobacterial toxin (cyanotoxin) toxicity can vary dramatically between different variants of the same toxin class (e.g., microcystins) and by exposure route (e.g., [25,26]). Of the limited studies that have examined the effects of inhaled cyanotoxins, the majority have assessed purified compounds such as microcystins, BMAA and anatoxin-a [19,25,27,28,29]. In terms of the real-world assessment of airborne toxins, different forms of environmental toxins, such as particles or free compounds, may have different toxicological effects in humans and animals. Regarding the many studies that have investigated cyanotoxins in air samples, the majority have employed collections on glass-fiber filter disks, isolating the algal filaments, colonies and cells from the air (e.g., [17]). An equally important scenario encompasses situations where blooms break down and release free toxins into the water, which can then be incorporated into LSA [30]. With the exception of cylindrospermopsin, which in culture can produce a significant extracellular pool of this toxin [31,32], most cyanotoxins (e.g., microcystins) are largely retained within cyanobacterial cells when the cells are healthy. Consequently, any air sampling scenario needs to consider these different compartments and the risks associated with exposure, such as with the use of impingers [16,20].

Southwest Florida is plagued by cyanobacterial blooms, including blooms of microcystin-producing *Microcystis* in eutrophic lakes such as Lake Okeechobee. Depending on environmental conditions and the season, large releases of toxic cyanobacteria from Lake Okeechobee to outflowing rivers can occur and result in aerosolized exposures in areas distant from the lake [33,34]. Such geographical displacement could lead to the potential for toxins, if made airborne, to also be transported long distances. When mass blooms of cyanobacteria occur, people often complain of many nonspecific symptoms such as breathing difficulties, coughs and allergies [35,36,37]. Therefore, gaining a greater understanding of the cyanotoxin types and concentrations in bloom components and air might help to explain such maladies. Studies elsewhere highlight the need for highly resolved temporal air measurements of cyanotoxins to better understand health risks [38], which requires the development of effective sampling devices that can be used for routine monitoring.

The aim of this study was to develop a portable air sampling device and examine the presence of particulate and free airborne cyanotoxins in Southwest Florida and compare the results of these analyses with water samples taken concurrently at the air sampling location. Ultimately, we aim to develop a repeatable, scalable operational tool to provide baseline data on the concentration of a range of cyanotoxins in Floridian waters with the goal of protecting human health and better understanding the airborne cyanotoxin exposure route.

## 2. Results

**Figure 1:**
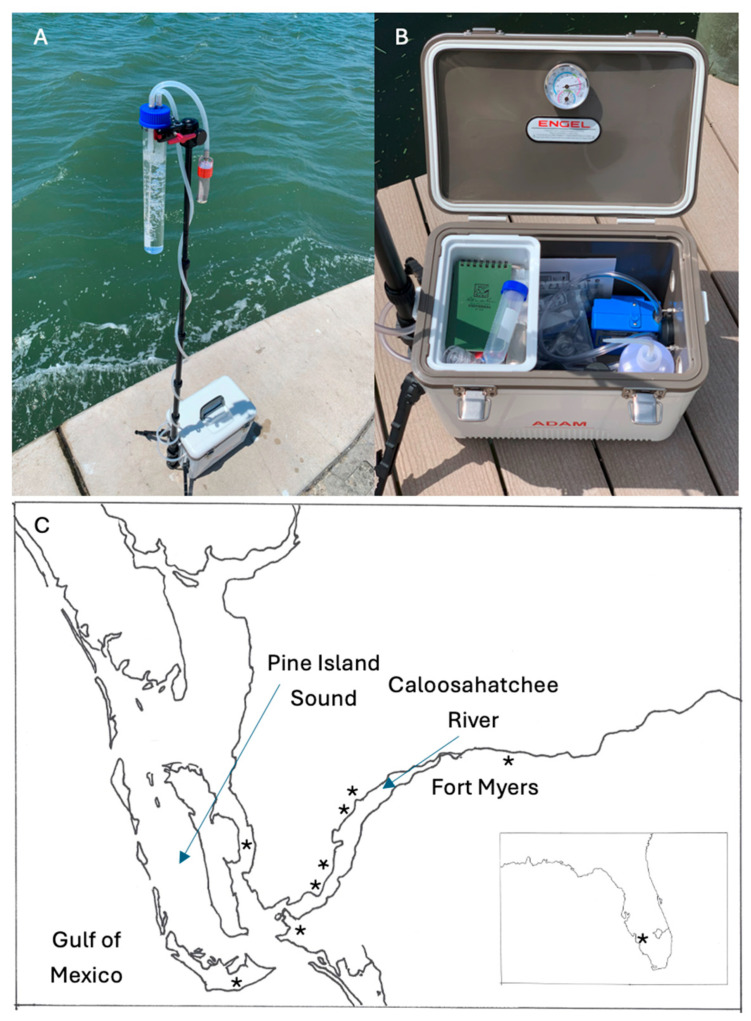
The ADAM showing the filter and impinger (**A**), along with the self-contained pump setup (**B**). (**C**) Location of air samplings (*).

A prototype air sampler was developed to assess the potential presence of cyanobacterial toxins and brevetoxin in air. Designed as a portable and robust single unit, each device consists of a waterproof field case with both battery and AC power capabilities, vent plugs to avoid air pump backpressure, and a collapsible monopole for lifting the system’s inflow to a representative human “breathing height” of between 5 and 6 feet (Figure 1A). The unit contains all components and supplies for transport from site to site with 10 min startups and shutdowns. Using a Sensidyne BDX-II air sampling pump (Figure 1B), toxins are captured with a Whatman 25 mm Glass Fiber Grade C (GF/C) filter (Cytiva, Global Life Sciences Solutions USA LLC, Wilmington, DE, USA) in series with a custom impinger (“water bubbler”) consisting of a 35 × 300 mm graduated hybridization tube (Chemglass LLC, Vineland, NJ, USA) with a dual port GL45 cap and a Chemglass course fritted 20 mm long gas dispersion tube acting through a 200 mL column of ultrapure water. While fiber filters have been traditionally used for aerosolized algal toxin capture, the addition of an impinger was more strategic: if this and other studies continue to prove the capture of airborne algal toxins by impingement, current and future rapid field-testing methods for algal toxins in source water can also be applied to impinger water. Weather conditions at sampling sites were typical for southwest Florida; variable weather was observed, but in general conditions were hot and humid, with frequent thunderstorms and coastal winds of 5–20 knots.

Of 21 water samples assessed, 13 (62%) did not show the presence of cyanobacteria or algae at the surface or near-surface of the water. As expected, in air samples, no cyanobacteria or algae were observed in impinger samples due to the placement of the air filter in-line before the impinger during operation. In water samples, one sample contained *Microcystis*, *Dolichospermum*, *Microcoleus* and diatoms, 6 other samples contained diatoms, and green algae were observed in 1 sample. However, with the exception of the *Microcystis* collection, most surface samples contained little in the way of observable cyanobacterial biomass according to light microscopy.

Using commercially available ELISA kits, cyanotoxins were observed in a number of samples at low concentration. ELISA kits assessed microcystins, cylindrospermopsins, anatoxin-a, saxitoxins and brevetoxins. Of these cyanotoxins, in filters (Table 1) only brevetoxin was detected in 5% of samples, with a mean low amount (1 pg per filter). In the impinger samples (Table 2), little of these cyanotoxins was detected, with anatoxin-a detected in 5% of samples, again at a very low amount. Concerning water samples collected at the same location as the air sample (Table 3), all 5 cyanotoxin classes were detected on occasion, with MC, CYN, ATXa, STX and Brevetoxin detected in 29, 29, 5, 43 and 38% of samples, respectively. Concentrations of these cyanotoxins were again generally low, consistent with the absence of noticeable surface blooms, with the exception of 1 sample that had a bloom of *Microcystis aeruginosa* and detectable microcystin-LR when analyzed by ELISA, UPLC-PDA and UPLC-MS.

Using LC-MS/MS, the presence and concentration of BMAA, AEG and DAB in samples was determined. For air filters (Table 1), these isomers were assessed as freely extractable compounds, a hydrolyzed free extract and in hydrolyzed solid material. As free compounds, AEG and BMAA were detected in 33 and 5% of samples, respectively, with 26 ng of AEG and 0.16 ng of BMAA present on the filter. In the hydrolyzed free fraction, all three isomers were detected with a higher frequency than for free compounds, although concentrations were similar. Hydrolysis of precipitable material showed only the presence of AEG and DAB, with lower concentrations of AEG than for the other fractions and DAB found at 6.2 ng per filter. In the impinger (Table 2), only free and hydrolyzed free fractions were analyzed as there was no precipitable material for extraction. In the free and hydrolyzed fractions, AEG, BMAA and DAB were more commonly found in 33, 29 and 52% of free samples, respectively, and at 100, 29 and 100% of the hydrolyzed samples, respectively. In general, the concentrations of these isomers in the impinger material were higher than in the filter material.

Although only one bloom of cyanobacteria was observed during the samplings, cyanotoxins and brevetoxin were commonly found at between 5 and 43% of samples, at generally low concentration, with the exception of MC-LR present at 15 μg/L by ELISA (Table 3) when a bloom of *M. aeruginosa* was analyzed. Analysis of the water samples showed that BMAA was not present in the free fraction, with or without hydrolysis, but was present in the hydrolyzed precipitable fraction in 38% of samples. The isomers of BMAA, AEG and DAB were found in surface water collections, although the concentration of all isomers was generally low, in the ng/L range.

Ultimately, although low amounts of anatoxin-a, microcystin and cylindrospermopsin were detected in air filters and impingers, BMAA and isomers were regularly detected.

## 3. Discussion

In order to help understand the occurrence of airborne cyanobacteria and their toxins in southwest Florida, along with the *Karenia brevis*-produced brevetoxin, which is commonly found in this area, an air sampling device was developed to standardize the collection of air samples for the assessment of a range of cyanobacterial and microalgal toxins in air, alongside on-site surface water collections. Although in the majority of sampling events, little to no visible cyanobacterial material was collected, low amounts of BMAA and isomers were frequently detected in air samples. Although counterintuitive, other studies examining airborne toxins have also found a lack of correlation between the amounts of cyanotoxins in air and in water or exposed sediment [13,16,20,39], and aerosols have the capacity to travel over 30 km from the affected source(s) [40]. Assessment of nasal swabs for microcystins from people who have had no recent water contact still found the presence of this toxin. However, assessment of nasal swabs from individuals who had direct contact with cyanobacterially contaminated water showed significantly higher MC concentrations than those who did not have direct contact [41], and data from a lung epithelial cell model suggested that lung cells exposed to microcystins could result in inflammation [28].

As a person breathes approximately 500 L of air per hour [42,43], it is possible that people who are not near a water source may still be chronically exposed to airborne cyanotoxins. Unlike most cyanotoxins, BMAA and isomers were commonly found in air samples according to triple quadrupole mass spectrometry, and, for example, airborne BMAA has previously been shown to be present at concentrations between 6 and 39 pg/L [19]. Shi et al. [44] demonstrated the presence of microcystin variants in aerosol samples from Grand Lake St. Marys in Ohio (USA) and found concentrations in pg m^−3^ in particulate matter from the lake (PM2.5). Furthermore, their study highlighted the importance of meteorological conditions concerning their release [44]. Although the majority of cyanotoxin analyses have examined air samples near waterbodies, largely to determine their presence in sprays and aerosols, terrestrial cyanobacteria may also release toxins into the air. The presence of cyanotoxins in desert crusts is well known [45,46], and in the case of the Great Salt Lake (Utah, USA), climate change and water management practices are contributing to the waters of this lake receding [47]. As a highly productive lake, with cyanobacteria being dominant organisms, including their presence as stromatolites on the lakebed, this loss of water is further exposing the lakebed, and wind action has resulted in the presence of BMAA and isomers as free compounds and associated with particles in air at low ng amounts [20].

Although a number of studies have examined the toxicological effect of single cyanotoxins through the intranasal or inhalation route (e.g., microcystin-LR, anatoxin-a [25]; microcystin-LR [27]; microcystin [28,48]) in environmental blooms of cyanobacteria, toxins are often present as mixtures, with co-occurrence and co-exposure to a cocktail of toxic compounds possible [8,49]. Under such a scenario, synergistic effects may occur [39,50] and nasally applied microcystin appears to have a 10-fold higher availability and toxicity than orally ingested toxins [48], suggesting that aerosolized toxins could represent a major risk for human populations close to lakes with cyanobacterial blooms. By analyzing a range of cyanobacterial toxins, the presence of multiple toxin classes in water [34,51,52,53,54] or multiple toxin variants (e.g., microcystins [55]; BMAA isomers [20]) in air samples demonstrates that providing an accurate risk assessment and guidelines to protect human health may be difficult.

The analyses of air samples in this study confirmed the findings of other studies that in the absence of significant blooms, cyanotoxins were still detectable in air samples and there was no correlation between the concentrations in air and water samples, with BMAA and isomers more frequently found than other classes of cyanotoxins. The ADAM device showed that baseline concentrations of toxins could be measured in air, as little bloom activity was observed in the vicinity of where sampling occurred at the time of collection. As the air sampling was performed over 24 h, future studies could employ more intensive water sampling to discount the possibility of vertical and horizontal bloom movement over that time.

The use of air samplers with a liquid impinger could also be adaptable to routine monitoring by directly analyzing the liquid on site using field test kits such as immunoassays. Such an approach with multiple sampling devices operating continuously could produce the high-resolution temporal data so desperately needed [38]. In the event of large-scale blooms, it would be expected that a person would likely be exposed to a greater concentration and variety of cyanotoxins than those who were not near contaminated waters. The ADAM device developed here provides a compact system that can look for the presence of a range of compounds with adverse health effects in both particulate and soluble fractions. For better human health risk assessment, standardized sampling protocols should be developed to allow greater comparison between studies. A greater understanding of the airborne cyanotoxin route of exposure is greatly needed to help protect human health and derive appropriate Guidelines for safe exposure levels.

## 4. Materials and Methods

### 4.1. Air and Water Sampling

Air samples were taken with an Airborne Detection for Algae Monitoring (ADAM) device (Figure 1A,B), a purpose-built air sampler to explore the scalability of aerosolized algal toxin detection for the extended geography and timeframe of this study. One or two secure sampling locations within the lower Caloosahatchee River and Matlacha Preserve areas were selected every two weeks. An ADAM device was run for 24 h in each selected location (e.g., Figure 1C, Supplementary Data Table S1) using an external electrical supply, given that the BDX-II battery lasts only eight hours. The placement of ADAM was typically on a dock or shoreline, above or no more than a few feet from the major water body. BDX-II flow rates were set at a “low flow” of 2 L per minute (LPM) at the start of sampling, and all cases were observed to have maintained this rate at shutdown, resulting in 2880 L of total air volume filtered. After shutdown, the GF/C filter cassette was sealed, impinger (Corning Incorporated, New York, NY, USA) water was transferred to a sample bottle, and a 250 mL sample of nearby source water was also taken at the same location for comparison. All samples were kept cold and express shipped to the lab for analysis [4].

### 4.2. Lab Processing of Samples

**Figure 2:**
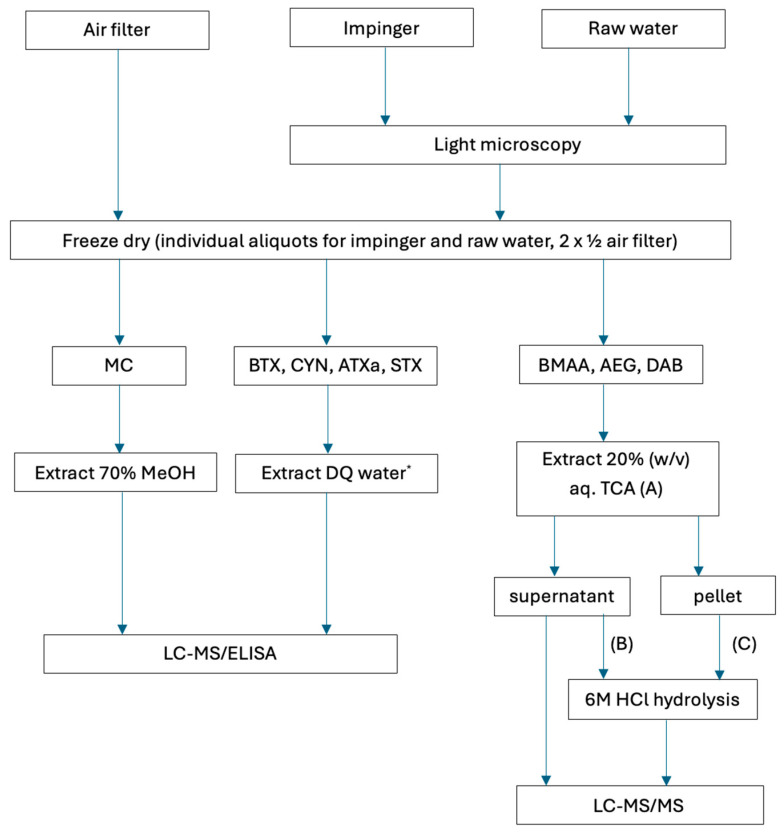
Schematic workflow for the processing of the environmental samples for toxin analysis. (A), free fraction; (B), hydrolyzed free fraction; (C), hydrolyzed fraction. *, raw water can be analyzed directly.

At each sampling time point, 3 sample types, a GF/C filter, approximately 20 mL of impinger liquid and a 250 mL water sample were assessed using a workflow developed specifically for the ADAM sampler (Figure 2). The impinger and water samples underwent light microscopy to observe the presence of any cyanobacteria and/or algae according to John et al. [56], and aliquots were then freeze-dried along with the filter disk before storage at −20 °C. Further liquid aliquots were stored at −20 °C for later assessment by ELISA.

Once freeze-dried, filters were warmed to room temperature and cut in half, with one half used for microcystin extraction and the other for extraction of BMAA and isomers. For microcystin extraction, the filter was cut into small pieces and resuspended with 70% (*v*/*v*) methanol, sonicated and left at room temperature for 1 h [57]. The extract was then stored at −20 °C overnight before warming back to room temperature. The methanol was removed from the filter material using a combination of centrifugation (12,000× *g*) and manual compression with a pipette tip, and the supernatant dried in a SpeedVac (Thermo Fisher Scientific, Waltham, MA, USA) and stored at −20 °C until analysis. For Ultra Performance Liquid Chromatography (UPLC) analysis, dried microcystin samples were resuspended with a minimal volume of 70% (*v*/*v*) methanol [4], centrifuged and the supernatant transferred to a sample vial.

The second half of the filter underwent extraction for BMAA and isomers [18,20,58]. The filter was warmed to room temperature and cut into small pieces. The pieces were then resuspended with 20% (*w*/*v*) trichloroacetic acid (TCA), sonicated and stored at 4 °C overnight. The following day, the samples were removed from 4 °C and warmed to room temperature. The supernatant was removed and placed in a fresh tube, again using centrifugation and manual compression. An aliquot of the supernatant was centrifuge-filtered, and the filtrate dried in the SpeedVac. A further aliquot underwent acid hydrolysis by addition of an equal volume of 12M HCl and heating at 110 °C for 16 h. The sedimented filter material was hydrolysed by addition of 6M HCl and heating at 110 °C for 16 h. The following day, the acid-hydrolysed samples were centrifuge filtered before drying of the filtrate in a SpeedVac and storage at −20 °C.

For water and impinger samples, a freeze-dried aliquot was used for MC analysis and a second freeze-dried aliquot was used for BMAA analysis. For microcystin analysis, a dried aliquot was resuspended in 1 mL 70% (*v*/*v*) methanol for analysis, sonicated and left overnight at −20 °C. The following day, the sample was warmed to room temperature, centrifuged and the supernatant transferred to a fresh tube, dried in a SpeedVac and stored at −20 °C. The BMAA aliquot was resuspended in a minimal volume of 20% (*w*/*v*) TCA, sonicated and stored overnight at 4 °C. The following day, the sample was warmed to room temperature and centrifuged. An aliquot of the supernatant underwent centrifuge filtration, and the filtrate was dried in a SpeedVac and stored at −20 °C. A further aliquot was mixed with an equal volume of 12M HCl and hydrolyzed at 110 °C for 16 h. The TCA extraction of the water sample also resulted in a visible pellet after centrifugation. This pellet was resuspended with 6M HCl and underwent acid hydrolysis for 16 h at 110 °C. The following day, aliquots were centrifuge filtered and the filtrate dried in the SpeedVac. All samples were then stored at −20 °C.

### 4.3. Analysis of Samples

Frozen aqueous aliquots of water and impinger samples were thawed, sonicated and centrifuged. The air filter MC extract (70% *w/v* methanol) was warmed to room temperature, resuspended with a minimal volume of 70% (*v*/*v*) methanol and diluted 1/10 with ultrapure water to give a rough methanol concentration of 7%, which did not adversely affect the ELISA. These samples were then assessed by commercial immunoassays for microcystin (Eurofins Abraxis, Warminster, PA, USA, P/N 520011), cylindrospermopsin (Eurofins Abraxis, Warminster, PA, USA, P/N 522011), anatoxin-a (Eurofins Abraxis, Warminster, PA, USA, P/N 520060), saxitoxin (Eurofins Abraxis, Warminster, PA, USA, P/N 52255B) and brevetoxin (Eurofins Abraxis, Warminster, PA, USA, P/N 520034) according to the manufacturer’s instructions (n = 2). The undiluted 70% (*v*/*v*) methanol microcystin extracts were also analyzed by UPLC-PDA and UPLC-MS (n = 3). For UPLC-MS and UPLC-PDA, the system consisted of a Waters Acquity Sample Manager, Binary Solvent Manager, column oven, PDA detector and QDA detector. Separation was achieved using a Phenomenex Kinetix 1.7 μm C18 column (100 × 2.1 mm i.d.; 00D-4475-AN) maintained at 40 °C with solvents of water + 0.1% formic acid (A) and acetonitrile + 0.1% formic acid (B) for MS analysis and water + 0.1% trifluoracetic acid (A) and acetonitrile + 0.1% (*v*/*v*) trifluoroacetic acid (B) for PDA analysis. Flow was delivered at 0.5 mL/min using a linear gradient of 30 to 70% B over 7 min, increasing to 100% B at 7.2 min for 0.8 min before returning to starting conditions at 8.2 min and a total run time of 12 min. Using a divert valve during mass spectrometric analysis, flow entered the QDa at 1.2 min and went to waste at 7.5 min. Seven commonly occurring microcystin variants were monitored as M+2H ions (MC-LA, m/z 455.75; D-Asp-MC-LR, m/z 491.27; demethyl-MC-LR, m/z 493.27; MC-LR, m/z 498.28; MC-LY, m/z 501.76; MC-RR, m/z 519.79; MC-YR, m/z 523.27; [59]) at a cone voltage of 6 V, capillary voltage of 0.8 kV, probe temperature of 400 °C and gain of 10. For the collection of PDA data, chromatograms were monitored at 238 nm with spectra obtained from 210 to 300 nm at a resolution of 1.2 nm in comparison with a MC-LR standard [34]. For BMAA analysis (n = 3), dried samples were resuspended with DQ water and derivatized with 6-aminoquinolyl-N-hydroxy-succinimidyl carbamate (AQC) and assessed by triple quadrupole mass spectrometry in comparison with AQC-derivatized standards of BMAA, N-(2-aminoethyl)glycine (AEG), β-amino-N-methyl alanine (BAMA) and 2,4-diaminobutyric acid (DAB) according to Banack [60].

## Figures and Tables

**Table 1:** Summary of the occurrence of cyanotoxins in air filters collected in southwest Florida.

Toxin	% Occurrence	Range (ng/Filter)	Mean (ng/Filter)
MC	0	ND	ND
CYN	0	ND	ND
ATXa	0	ND	ND
STX	0	ND	ND
BTX	4.8	ND-0.01	0.001
Free AEG	33.3	ND-26.08	5.47
Free BMAA	4.8	ND-0.16	0.008
Free DAB	0	ND	ND
Free hydrolyzed AEG	100	0.04–12.48	2.37
Free hydrolyzed BMAA	14.3	ND-0.82	0.039
Free hydrolyzed DAB	4.8	ND-0.12	0.006
Hydrolyzed AEG	100	NQ-0.64	0.14
Hydrolyzed BMAA	0	ND	ND
Hydrolyzed DAB	28.6	ND-6.20	0.57

MC, microcystin; CYN, cylindrospermopsin; ATXa, anatoxin-a; STX, saxitoxin; AEG, N-(2-aminoethyl)glycine; BMAA, β-*N*-methylamino-L-alanine; DAB, 2,4-diaminobutyric acid; ND, not detectable; NQ, not quantified.

**Table 2:** Summary of the occurrence of cyanotoxins in impingers collected during air sampling in southwest Florida.

Toxin	% Occurrence	Range (ng/Sample)	Mean (ng/Sample)
MC	0	ND	ND
CYN	0	ND	ND
ATXa	4.8	ND-0.008	0.0004
STX	0	ND	ND
BTX	0	ND	ND
Free AEG	33.33	ND-3.7	0.52
Free BMAA	28.6	ND-2.5	0.26
Free DAB	52.4	ND-3.2	0.51
Free hydrolyzed AEG	100	NQ-24.0	5.94
Free hydrolyzed BMAA	28.6	ND-3.4	0.60
Free hydrolyzed DAB	100	NQ-38.0	3.39

MC, microcystin; CYN, cylindrospermopsin; ATXa, anatoxin-a; STX, saxitoxin; AEG, N-(2-aminoethyl)glycine; BMAA, β-*N*-methylamino-L-alanine; DAB, 2,4-diaminobutyric acid; ND, not detectable; NQ, not quantified.

**Table 3:** Summary of the occurrence of cyanotoxins in water samples collected at the time of air sampling in southwest Florida.

Toxin	% Occurrence	Range (μg/L)	Mean (μg/L)
MC	28.6	ND-15.39	0.77
CYN	28.6	ND-0.08	0.02
ATXa	4.8	ND-0.22	0.01
STX	42.9	ND-0.32	0.04
BTX	38.1	ND-0.66	0.05
		**Range (ng/L)**	**Mean (ng/L)**
Free AEG	66.7	ND-80.00	3.81
Free BMAA	0	ND	ND
Free DAB	9.5	ND-52.1 *	4.01 *
Free hydrolyzed AEG	100	ND-7.5	0.36
Free hydrolyzed BMAA	0	ND	ND
Free hydrolyzed DAB	71.4	ND-400	75.23
Hydrolyzed AEG	81.0	ND-2.6	0.85
Hydrolyzed BMAA	38.1	ND-22.0	2.03
Hydrolyzed DAB	100	2.1–316.5	51.86

MC, microcystin; CYN, cylindrospermopsin; ATXa, anatoxin-a; STX, saxitoxin; AEG, N-(2-aminoethyl)glycine; BMAA, β-*N*-methylamino-L-alanine; DAB, 2,4-diaminobutyric acid; ND, not detectable; NQ, not quantified; *, concentrations reported as ug/L.

## Data Availability

The original contributions presented in this study are included in the article/Supplementary Materials. Further inquiries can be directed to the corresponding author.

## Supplementary Materials

The following supporting information can be downloaded at: https://www.mdpi.com/article/10.3390/toxins18070309/s1, Table S1: Environmental information and location of air sampling.
