# Supplementary material for: Developing Tools to Assess Airborne Cyanobacterial Toxins in Southwest Florida, USA
Source: Toxins (Basel). 2026 Jul 16;18(7):309. doi: 10.3390/toxins18070309 (PMC13417131; doi:10.3390/toxins18070309)
Supplement: Supplementary file 1 [file toxins-18-00309-s001.zip › toxins-4375049-supplementary.pdf]

# Supplementary Materials: Developing Tools to Assess Airborne Cyanobacterial Toxins in Southwest Florida, USA

**James S. Metcalf, Manuel Aparicio, Sandra A. Banack, Jason Pim, John R. Cassani and Paul A. Cox**

**Table S1:**

| Sample ID | Date | Location | Duration HRs. | start time | Liters per minute | Bubbler vol. ml to start | Comments | Wind speed |
|---|---|---|---|---|---|---|---|---|
| BCL-001 | 7/13/21 | 26.579730, -81.940626 | 24 | 9:00 | 1.5 | 20 | Pearl Canal (tidal Cape Coral canal) Thunderstorms with wind and rain during early afternoon on 7/13/21. Intermittent red tide since November 2020, approximately seven miles to the west in San Carlos Bay, Lee County. Salinity at the mouth of the Caloosahatchee was 13 psu on low tide and 30 on high tide. The tidal canal would reflect this variation but might have had lower salinity on low tide due to area rainfall and runoff. Will record on-site salinity with the next sample. The area also had intermittent Dapis (Lyngbya syn.). No cyanobacteria was observed on the surface despite the same canal having very high levels in July 2018. 99%+ of the Caloosahatchee outflow has been from basin runoff, not Lake Okeechobee over the past 10 days or so. | x |
| BCL-002 | 7/20/21 | 26.643063, -81.905270 | 24 | 9:00 | 2.0 | 40 | Sunny and warm. Wind and other field conditions forthcoming. Red tide conditions in Lee County absent or very low. The shallow edge of the river about 50 feet from the aerosol sampler had a small area of what appeared to be | x |

| | | | | | | | | |
|---|---|---|---|---|---|---|---|---|
| | | | | | | | microcystis at the surface and intermittent filamentous algae (cyanobacteria) similar to Lyngbya or Dapis. Both types of algae or cyanobacteria were included with the BCL-002-S sample. Note image attached. Used 2.0 um pore size bubbler | |
| BCL-003 | 7/27/21 | 26.721306, -81.721026 | 24 | 9:00 | 1.7-1.75 | 30 | Upper Caloosahatchee estuary waterfront home. no visually discernible cyanobacteria or algae, heavy overnight rain. | x |
| BCL-004 | 7/27/21 | 26.63438, -82.065752 | 24 | 9:00 | 1.5-2.0 | 30 | Stilt house on Matlacha Pass near Matlacha Bridge. no visually discernible cyanobacteria or algae, periodic rain | x |
| BCL-005 | 8/10/21 | 26.63438, -82.065752 | 24 | 9:00 | 2.0 | 40 | Stilt house (Ms. Williams home) on Matlacha Pass, browning look to the water, shifted to 5.0 um airstone hereafter | x |
| BCL-006 | 8/10/21 | 26.643063, -81.905270 | 24 | 9:00 | 2.0 | 40 | Same NFM location on the Caloosahatchee estuary | |
| BCL-007 | 8/17/21 | 26.721306, -81.721026 | 24 | 9:00 | 1.5-2.0 | 40 | Upper Caloosahatchee estuary waterfront home. No visual aglae present | |
| BCL-008 | 8/17/21 | 26.276180, -81.863579 | 24 | 9:00 | NR | 40 | Caloosahatchee estuary, Old Bridge Village, waterfront | |
| BCL-009 | 9/1/21 | 26.721306, -81.721026 | 24 | 9:00 | 2.0 | 30 | Upper Caloosahatchee estuary waterfront home. No visual aglae present, bubbler evaporating to 20 ml, 70-100 um impinger first use of this bubbler | x |
| BCL-010 | 9/1/21 | 26.512588, -81.998688 | 24 | 9:00 | 2.0 | 30 | Manny's House, West side of Shell Point, Punta Rassa Cove off of Shell Creek, high turbidity in River, evaporating to 15 ml, 70-100 um impinger | x |
| BCL-011 | 9/8/21 | 26.721306, -81.721026 | 24 | 9:00 | 2.0 | 30 | No algae in River observed, evaporated to 20 ml, 70-100 um impinger | x |
| BCL-012 | 9/8/21 | 26.643063, -81.905270 | 24 | 9:00 | 2.0 | 30 | 85 degrees, 5 knot SW wind, evap to 15 ml, 70-100 um impinger | 5 |
| BCL-013 | 9/22/21 | 26.4243, -82.0656 | 24 | 9:00 | 2.0 | 30 | evap. To 20 ml, | |

| | | | | | | | | |
|---|---|---|---|---|---|---|---|---|
| BCL-014 | 10/6/21 | 26.276180, - 81.863579 | 24 | 9:00 | 2.0 | 30 | | |
| BCL-015 | 10/6/21 | 26.553606, - 81.945769 | 24 | 9:00 | 2.0 | 30 | | |
| BCL-016 | 10/27/21 | 26.4243, - 82.0656 | 24 | 9:00 | 2.0 | 30 | | |
| BCL-017 | 10/27/21 | 26.643063, - 81.905270 | 24 | 9:00 | 2.0 | 30 | | |
| BCL-018 | 11/9/21 | 26.512588, - 81.998688 | 24 | 9:00 | 2.0 | 30 | wind NE at 10 mph, 85 degress, 82% humidity | 10 |
| BCL-019 | 11/9/21 | 26.276180, - 81.863579 | 24 | 9:00 | 2.0 | 30 | | |
| BCL-020 | 11/30/21 | 26.721306, - 81.721026 | <24 | 9:00 | 2.0 | 30 | pump wasn't running on arrival the following day, connector was disconnected | |
| BCL-021 | 11/30/21 | 26.643063, - 81.905270 | 24 | 9:00 | 2.0 | 30 | | |
| NR, not recorded | | | | | | | | |
